# Factors influencing transitional care from adolescents to young adults with cancer in Taiwan: A population-based study

**DOI:** 10.1186/s12887-016-0657-z

**Published:** 2016-08-02

**Authors:** Yo-Ting Jin, Chin-Mi Chen, Wu-Chien Chien

**Affiliations:** 1 The Graduate Institute of Medical Sciences, National Defense Medical Center, Taipei, Taiwan; 2 Department of Nursing, Fu Jen Catholic University, Taipei, Taiwan; 3 Department of Medical Research, National Defense Medical Center, Tri-Service General Hospital, Taipei, Taiwan; 4 School of Public Health, National Defense Medical Center, Taipei, Taiwan

**Keywords:** Transitional care, Adolescent, Cancer, Children with special health care needs

## Abstract

**Background:**

To investigate the progress of transition from paediatric to adult health care for patients with cancer in Taiwan’s medical system.

**Methods:**

The data were retrieved from the Longitudinal Health Insurance Database (LHID), which contains the original inpatient and outpatient medical claims data for 1,000,000 enrollees randomly sampled from the NHIRD between 1997 and 2010.

**Results:**

Among the 1,411 cancer patients selected for this study, 98.09 % received adult-oriented therapy before the age of 18. In addition, only 1.91 % of the patients received paediatric-oriented therapy during adolescence. The primary factors that determine whether these patients would receive paediatric-oriented therapy or adult-oriented therapy at an early age were as follows: the age of the patient at the first visit and the performance-level of the hospital (*p* < 0.001).

**Conclusions:**

Previous studies conducted in developed countries have demonstrated that the unwillingness of patients to switch from paediatric-oriented therapy to adult-oriented therapy being the major obstacle that hinders the transition process. However, this study revealed a different result: the implementation of the National Health Insurance system in Taiwan makes healthcare affordable for the adolescent patients who may not possess adequate knowledge about paediatric health care and may not appreciate paediatric-oriented therapy, thereby hindering the transition process.

## Background

In recent years, with the significant advances in medical science and technology, more children with chronic diseases can survive into adulthood [1–3]. Childhood cancer in particular exhibits a high survival rate of approximately 80 % [4]. Consequently, when transitioning from paediatric to adult healthcare services, these children, their families, and healthcare providers may encounter numerous potential problems, such as adult health insurance, adaptation to adult life, and financial conditions that create specific healthcare needs [5–9], because the healthcare environment for children must be changed from dependent to independent [10–12].

Transitional care is widely defined as “the purposeful, planned movement of adolescents and young adults with chronic physical and medical conditions from child- centered to adult-oriented healthcare systems” [13]. An effective transition process can provide appropriate, high-quality, and uninterrupted medical care services for the patient, as well as a communication platform for the main participants in the patient’s treatment, including the patient, family members, paediatricians, nurses, adult-healthcare providers, and other healthcare professionals [14–22], to enhance the patient’s health, life outcomes, self-management and autonomy [23, 24].

In developed western countries such as the United Kingdom and Australia, the transitional medicine is supported by the government and has been implemented for years [25]. In United States, some individual states and health services have recognised the importance of transition and the need for formal approaches to transition planning [26]. Numerous researchers have demonstrated that the bond formed among the patients, their families, and adult-healthcare providers during transitional care provides excellent results, including reduced length of stay [27], reduced medical costs [28], and increased medical usage satisfaction of adolescent patients with chronic illnesses and their families [29, 30].

**Fig. 1:**
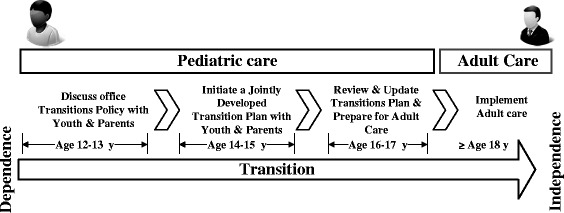
A Transition Process from Paediatric to Adult Services

All previous research about the transition from paediatric care to adult health care has suggested that timing is critical for a successful transition. A transition process for all youth has been provided [31], as shown in Fig. 1. Age is one of the most frequently discussed predictors of the transition time decision [32]. Although no agreement has been reached on the appropriate age of transitional care in previous studies, numerous studies have indicated that an appropriately timed transition from a paediatric-oriented to an adult-oriented clinic typically should occur between 18 and 19 years of age [33–35]. Moreover, several obstacles to successful transitional care programs have been identified in the past decade, and these can be mainly divided as follows: unwillingness to leave familiar medical professionals and environments [32], unable to adapt to adult-oriented medical care [32], poor timing of transition [26], lack of adult medical services [36], lack of proper health insurance [4, 32], and absence of transitional care knowledge [37]. Particularly in continue having proper health insurance, another age-related concern, patients are discouraged to engage in their follow-up mainly due to lack of insurance as they grow up and become too old to participate in parental/public insurance and lack of fulltime employment [38, 39]. Also, insurance policies often have restricted listings of contracted physicians, and finding someone with the willingness and expertise to follow up with childhood cancer survivors may be difficult [40].

**Fig. 2:**
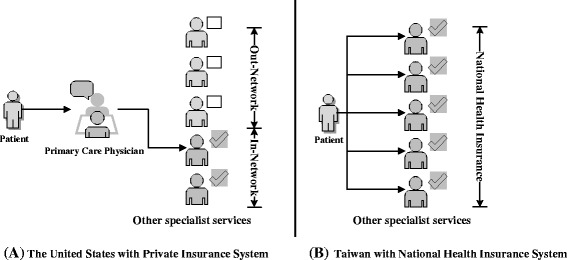
The comparison of Health Insurance and Health care system between Taiwan and U.S.

Cultural and medical policies can also influence the implementation results or create new obstacles to transitional care. As shown in Fig. 2, for example, compared with the insurance and health care system in the United States, Taiwan’s National Health Insurance allows unrestricted access to physicians of all specialties irrespective of age and personal finance. Therefore, lack of proper health insurance is not an obstacle transitioning from paediatric to other adult-oriented services. However, transition care services have not been completely established in Taiwan. It is difficult for patients to seek an appropriate specialist or medical service without the help of their primary care physicians if the patients lack medical knowledge. Moreover, only two studies related to transitional care in Asia have been conducted. Wong et al. [41] and Ishizaki et al. [42, 43] have investigated transitional care in Hong Kong and Japan, respectively, and the results indicated that both countries lack an established transition program from paediatric to adult healthcare services.

The goals of this study are to identify the characteristics of childhood cancer patients, and to investigate the progress of transition from paediatric to adult health care for these patients in Taiwan’s medical system. The Taiwanese government initiated a single-payer National Health Insurance programme approximately 20 years ago; Taiwan’s National Health Insurance system covers most forms of treatment, including general diagnoses and treatment, medical consultations and operations, and other related procedures such as examinations, laboratory tests, anaesthesia, prescription medications, supplies, nursing care, hospital admissions, and certain over-the-counter drugs. A family of four pays a premium of roughly United States Dollar (USD) $100 per month, accounting for about 2 % of the average household income. In addition, over 23 million people were enrolled in the National Health Insurance Research Database (NHIRD) after implementing the programme, thus granting researchers access to a large amount of samples for analysis. Compared with most previous studies on transitional care, which used questionnaire methods to determine the needs of a small number of patients, families, medical professionals, and adult-healthcare providers by interviewing them [32], the analytic results derived from a larger sample from the NHIRD are more reliable and more representative.

## Methods

### Data source and sample

In this study, the data were retrieved from the Longitudinal Health Insurance Database (LHID) 2005, which contains the original inpatient and outpatient medical claims data for 1,000,000 enrollees randomly sampled from the NHIRD between 1997 and 2010. We selected patients diagnosed with neoplasms before the age of 18 years based on the International Classification of Diseases, Ninth Revision (ICD-9) codes 140–239 and maintained an adult electronic medical record in the LHID 2005.

#### Variables related to healthcare transition

##### Dependent variables

The Childhood Cancer Survivor Study revealed a phenomenon that approximately a third of young adult survivors experiencing difficulty in obtaining health insurance beginning at 18 years of age [39]. In comparison, the siblings of these young adults or those around the same age who are in good health do not share the same experience. In addition, 18 is the legal age of adulthood in Taiwan. Therefore, this study used the following criterion to identify patients who meets a well-timed transition: if a patient received paediatric services before the age of 18 years and adult-healthcare services in adulthood, a successful transfer was achieved; otherwise, no transitional care was received.

##### Independent variables

In previous studies, patient characteristics and characteristics of medical service providers were common explanatory variables used to identify factors and barriers associated with transition decision. In this study, we used the performance-level of hospitals where patients visited prior to adulthood, and hospital location, the criteria obtained from the registration files of the LHID 2005, as the characteristics of medical service providers. The hospital performance-levels were assigned by a professional Taiwanese medical evaluation institution by performing objective assessments of the quality of medical services provided. Hospitals were categorized into one of the following three categories: academic medical centers (AMCs); superior in hospital accreditation and excellence in teaching hospital (SHET); and others. AMCs and SHET represent hospitals with the highest level of medical service quality according to the old and new evaluation systems, respectively. AMCs takes a more holistic approach in providing care to patients, including medical education and research, development of medical and health technology. In contrast, SHETs are based on patients’ point of view and strive to provide all the relevant information to the patients’ condition prior to giving any medical treatment. In other word, AMCs generally focus on teaching life-saving skills, whereas SHETs not only focus on teaching life-saving skills, but also on teaching the purpose and the quality of the care provided. Hospital locations were divided into six major cities of Taiwan and non-metropolitan regions based on the area classification of Taiwan. These major cities included Taipei, New Taipei, Taoyuan, Taichung, Tainan, and Kaohsiung. Urban regions were defined as areas with a population density of at least 1,500 people per square kilometer, and rural regions were defined as those with 1,499 or fewer people per square kilometer. The following information for patient characteristics was retrieved from the database: gender, average healthcare expenses and yearly clinical visit frequency before 18 years of age, cancer, and the age of first visit. Cancer was divided into single and multiple primary cancer types. Single primary cancer denotes patients with only one type of cancer, whereas multiple primary cancer represents patients with other complications as a result of the cancer(s) and other patients not classified in the single primary cancer category. Average medical expenses and clinical visit frequency both were divided into low and high level based on the average.

#### Statistical analyses

Frequency analysis, comprising *t* tests for continuous data and multivariate logistic regression models for determining the independent contributions of explanatory variable to the appropriateness of transition time, was conducted in this study. A significance level of α = 0.01 was established to determine the significance of the results. Microsoft Access 2010 was used to filter all data and IBM SPSS 20.0 was employed to perform all statistical analyses.

## Results

A total of 1,411 patients were selected for this study, and the characteristics of the sampled patients are summarized in Table 1. The subjects were aged 18–29-years, with a mean age of 20.97 years. 946 were female and 465 were male. There were 34.66 % who had simple primary cancer. The majority of the patients received their first therapy in LHID between 15 and 18 years of age (75.97 %), followed by 12–15 years of age (18.50 %); only 0.85 % of the patients age below 9 years of age. Hospital location distribution was 17.51 % Taipei, 5.67 % New Taipei, 9.64 % Taoyuan, 6.87 % Taichung, 4.11 % Tainan, 10.84 % Kaohsiung, and 45.36 % Others. Hospital performance-level was 3.97 % AMCs, 22.18 % SHET and 73.85 % others. A total of 78.10 % of patients received care at clinics 0–2 times per year on average before the age of 18 years. With respect to medical expenses, 53.08 % of patients incurred an average expenditure of USD $3 or less per visit before 18 years of age. The mean medical expense for receiving care at pediatric clinics was USD $2.7 per visit, while the mean medical expense for receiving care at non-pediatric clinics was USD $3.65 per year. The percentage of patients who incurred an annual expenditure of US $3.65 or less per visit on adult-oriented medical expenses was 55.36 %; 3.05 % of these patients incurred an expenditure of US $2.7 or less per visit on paediatric-oriented medical expenses; and 94.61 % of these patients had never receive paediatric-oriented therapy. For many patients, the first time they received adult-oriented therapy was between the ages of 15 and 18 years old (75.69 %). Another 22.40 % of patients received adult-oriented therapy before the age of 15. The age for which patients received adult-oriented therapy (mean = 16.03, SD = 1.99) was significantly less than 18 (*p* < 0.001). Moreover, the frequency of yearly clinic visits for patients receiving transition cares was (Mean = 1.81, SD = 1.45, *n* = 15) significantly lower than that of patients not receiving transition cares (Mean = 3.73, SD = 15.79, *n* = 1,396; *p* = 0.001).

**Table 1:** Sample Characteristics of Participants (*N* = 1,411)

Character	Observation (*n*)	Ratio (%)	Mean	Standard Deviation (SD)
Age (range 18–29 years)			20.97	2.30
Gender				
Female	946	67.04 %		
Cancer				
Simple primary	489	34.66 %		
Multiple primary	922	65.34 %		
Hospital				
AMCs	56	3.97 %		
SHETs	313	22.18 %		
Others	1042	73.85 %		
Area				
Taipei	247	17.51 %		
New Taipei	80	5.67 %		
Taoyuan	136	9.64 %		
Taichung	97	6.87 %		
Tainan	58	4.11 %		
Kaohsiung	153	10.84 %		
Others	640	45.36 %		
Age at first visit			15.91	2.00
<9	12	0.85 %		
9–12	66	4.68 %		
12–15	261	18.50 %		
15–18	1072	75.97 %		
Clinic visit frequency (mean yearly) before age of 18			2.02	3.76
Low (≤2 times)	1102	78.10 %		
High (> 2 times)	309	21.90 %		
Mean medical expenses per visit before age of 18			3.00	2.03
Low (≤ USD $ 3)	749	53.08 %		
High (> USD $3)	662	46.92 %		
The medical expenses of adult service ($/time)			3.65	2.23
Low (≤ USD $ 3.65)	770	55.36 %		
High (> USD $ 3.65)	629	45.22 %		
No receiving adult service	12	0.86 %		
The medical expenses of pediatric service ($/time)			2.70	3.12
Low (≤ USD $ 2.7)	43	3.05 %		
High (> USD $ 2.7)	33	2.34 %		
No receiving pediatric care	1335	94.61 %		
Age at first receiving adult service				
<12	67	4.75 %	16.03	1.99
12–15	249	17.65 %		
15–18	1068	75.69 %		
18–21	11	0.78 %		
≥21	4	0.28 %		
No receiving adult service	12	0.85 %		

**Fig. 3:**
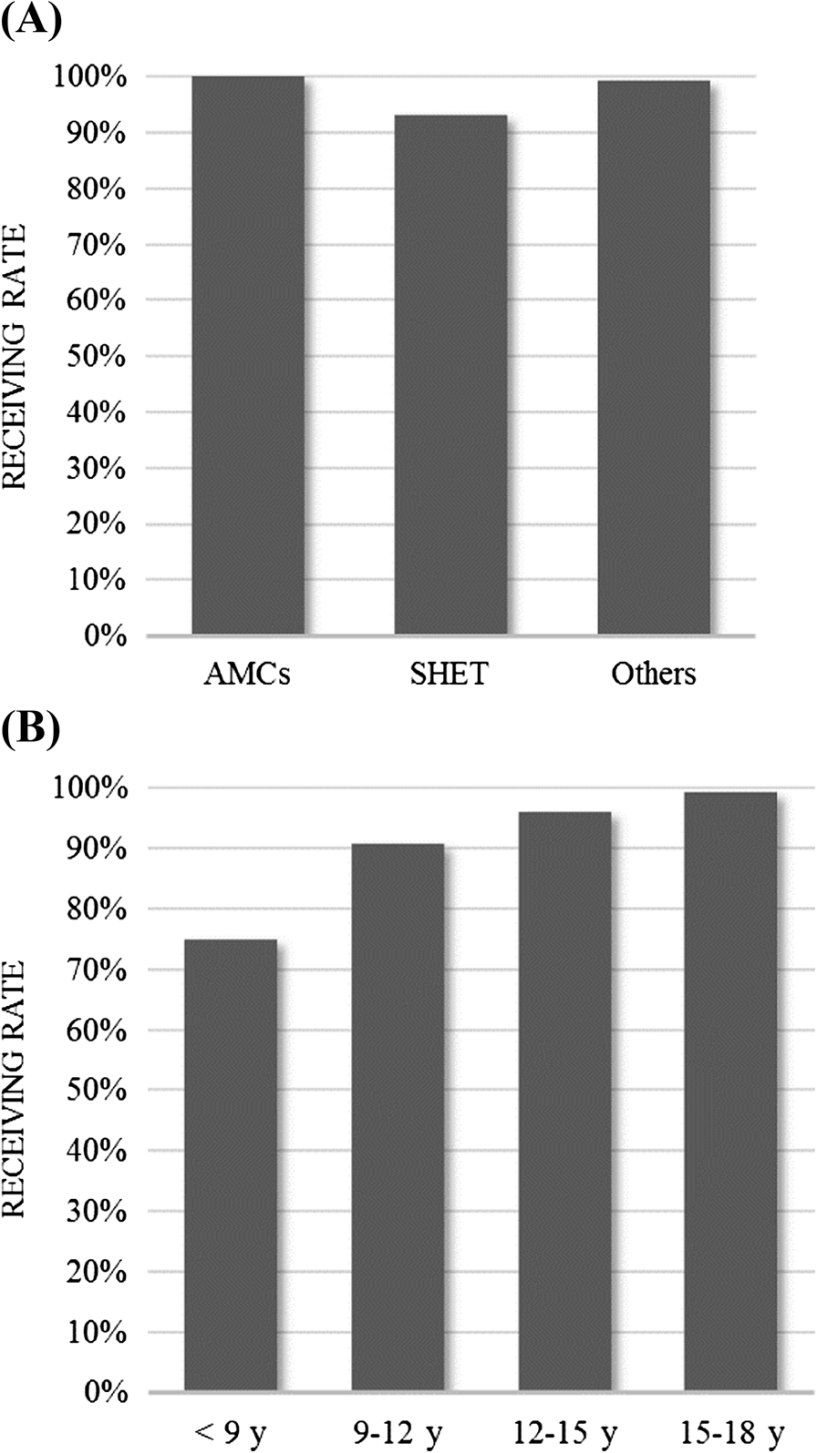
Significant Factors Related to The Percentage of Patients Receiving Care in Pediatric Health Care Clinics Before the Age of 18 Years. **a** Hospital performance-level. **b** The age of first visit

Table 2 shows the factors associated with patient visits to adult-oriented care before the age of 18 years. In multivariate logistic regression, SHET (OR 9.044 [2.840, 28.807], *β* = 2.202, *p* < 0.001) were positively associated with patient visits to adult-oriented care before the age of 18 years. Figure 3a shows that the rate of prematurely receiving adult-oriented care in hospitals accredited with SHET accounted for 93.29 %, lower than that of hospitals of AMCs and other performance-levels (100 and 99.42 %, respectively). The age of first visit (OR 0.738 [0.647, 0.842], *β* = −0.304, *p* < 0.001) was inversely associated with patient prematurely receive adult-oriented care. As shown in Fig. 3b, the rate of prematurely receiving adult-oriented care was 75 % in ≤9 years of age, 90.91 % in 9–12 years of age, 96.17 % in 12–15 years of age, and 99.25 % in 15–18 years of age. Other factors, including gender, cancer, hospital location, mean medical expenses per visit, and mean clinic visit frequency were not associated with patient visits to paediatric healthcare clinics before the age of 18 years.

**Table 2:** Factors Associated with Receiving Care at Adult Health Care Clinics Before the Age of 18 Years on Multiple Logistic Regression Analysis

Variables	Multivariate logistic regression			
	*β*	Odds Ratio (OR)	*95 % CI*	*p* value
Gender				
Female	−0.844	0.430	0.184–1.005	0.051
Male	Reference			
Cancer				
Simple primary	0.173	1.189	0.486–2.905	0.705
Multiple primary	Reference			
Mean medical expenses per visit before age of 18				
Low (≤ USD $3)	0.912	2.489	1.020–6.077	0.045
High (> USD $3)	Reference			
Clinic visit frequency (mean yearly) before age of 18				
Low (≤2 times)	0.130	1.139	0.394–3.295	0.810
High (>2 times)	Reference			
The age of first visit	−0.304	0.738	0.647–0.842	< 0.001
Hospital location				
Taipei	−0.937	0.392	0.071–2.171	0.284
New Taipei	0.165	1.179	0.260–5.352	0.831
Taoyuan	−16.151	0.000	0.000 - .	0.997
Taichung	−0.242	0.785	0.137–4.501	0.786
Tainan	0.967	2.630	0.506–13.665	0.250
Kaohsiung	−15.769	0.000	0.000 - .	0.997
Non-metropolitan	Reference			
Hospital Performance-level				
AMCs	−15.405	0.000	0.000 - .	0.997
SHETs	2.202	9.044	2.840–28.807	< 0.001
Others	Reference			

Number of patients in model = 1,411; Nagelkerke *R* ^2^ = 0.312

## Discussion

Previous studies have demonstrated that when insurance systems does not cover the medical expenses for adult health care, adolescent patients will prolong the period of paediatric health care [32]. In Taiwan, people can receive medical treatment at a low cost regardless of the type of care and the limitation of age because of National health insurance system. The mean medical expenses per visit is less than USD $3. The financial burden borne by patients with cancer is less severe, and patients are able to switch from paediatric to adult health care without contemplating future medical expenses. Instances of delayed transition to adult care are relatively rare. Thus, the mean medical expense was not a significant barrier in the regression.

However, the vast majority of the patients begin receiving adult health care well before the age of 18 and few adolescent patients with cancer are provided a complete transition mechanism. In fact, the expenses of adult-oriented health care are higher than those of pediatric health care (*p* = 0.01). But, patients’ families are likely to favor adult-oriented therapy when choosing the medical facilities for their adolescent children. This would jeopardize the mental and physical development of children with cancer. Moreover, transition planning requires long-term preparation. Premature transition to adult-oriented therapy may lead to insufficient preparation, resulting in transition failure^7^.

Taiwan Pediatrics Association has completed a survey of 14,730 parents in Taiwan via a web questionnaire. The results show that 83 % of parents don’t know their children should receive pediatric-oriented therapy before age of 18 and 58.5 % of parents don’t know which department their children should visit. In other words, the fact that patients and their families lack the knowledge related to pediatric health care could be the reason why patients did not receive any pediatric-oriented therapy or accepted adult-oriented therapy early. In Taiwan, most hospitals have a paediatric specialist that can treat those patients and hold a consultation with other specialists if necessary. But, hospitals accredited with the SHET have more complete healthcare systems and provide more comprehensive health education than do hospitals of other performance-levels. Patients receiving health care in these hospitals are more favourably positioned to learn about paediatric health care. The ratio of patients prematurely receiving adult health care at hospitals accredited with the SHET was significantly lower than that of patients receiving care at hospitals of other performance-levels, which again well demonstrated that transition failure is due to patients’ lack of understanding of the influence of paediatric health care on adolescent patients, the lack of a transition mechanism in hospitals, and hospitals failing to provide related information to patients and their families. Therefore, patients and their family members choose their health care based solely on the department’s specialty (e.g., patients with cancer choosing the department of oncology), resulting in transition failure. In Taiwan, adolescent survivors and their family should be informed about the paediatric healthcare and the supporting groups that can assist in transitioning care.

In this study, the physicians’/pediatricians’ views on transition care were not explored because the LHID contains only the circumstantial factors, such as medical expenses, hospital location and hospital performance-level. However, the scope of the investigation has provided a relevant depiction of the effect of a low-cost health insurance system have on transitional care. A starting point has been provided by this study for factors to transitional care in areas that have implemented national health insurance.

## Conclusion

As the survival rate of adolescent patients with cancer continues to rise, transition mechanisms also become increasingly vital. Transition mechanisms provide patients with appropriate health care as their demands change when transitioning from adolescence to adulthood. These mechanisms enhance their quality of life and reduce medical expenses. While researchers in numerous developed western countries have looked into various topics surrounding transitional care in recent years, in Asia, only researchers in Japan and Hong Kong have conducted similar research. These studies indicated that health insurance and unwillingness to leave familiar medical professionals and environments were the two common factors contributing to transition failure.

However, factors influencing the transition care differ between cultures and health policies. In this study, we conducted investigations to explore the situation of transition care for Taiwan, where the National Health Insurance system has been implemented. The major transition obstacles hindering the transition from paediatric to adult health care in Taiwan differ substantially from those suggested by previous studies. In Taiwan, the adolescents with cancer have a very short period of paediatric treatment or have never receive paediatric treatment. Lack of appropriate paediatric treatment is the very obstacle for successful transitional care.

## Abbreviations

AMCs, Academic Medical Centers; LHID, longitudinal national health insurance research database; NHIRD, National Health Insurance Research Database; OR, odds ratio; SD, standard deviation; SHET, Superior in Hospital accreditation and Excellence in Teaching hospital; USD, United States Dollar.
